# Decreased transcription-coupled nucleotide excision repair capacity is associated with increased p53- and MLH1-independent apoptosis in response to cisplatin

**DOI:** 10.1186/1471-2407-10-207

**Published:** 2010-05-14

**Authors:** Lawton J Stubbert, Jennifer M Smith, Bruce C McKay

**Affiliations:** 1 Cancer Therapeutics Program, Ottawa Hospital Research Institute, Ottawa, ON, Canada; 2 Department of Medicine, University of Ottawa, Ottawa, ON, Canada; 3 Department of Cellular and Molecular Medicine, University of Ottawa, Ottawa, ON, Canada

## Abstract

**Background:**

One of the most commonly used classes of anti-cancer drugs presently in clinical practice is the platinum-based drugs, including cisplatin. The efficacy of cisplatin therapy is often limited by the emergence of resistant tumours following treatment. Cisplatin resistance is multi-factorial but can be associated with increased DNA repair capacity, mutations in p53 or loss of DNA mismatch repair capacity.

**Methods:**

RNA interference (RNAi) was used to reduce the transcription-coupled nucleotide excision repair (TC-NER) capacity of several prostate and colorectal carcinoma cell lines with specific defects in p53 and/or DNA mismatch repair. The effect of small inhibitory RNAs designed to target the CSB (Cockayne syndrome group B) transcript on TC-NER and the sensitivity of cells to cisplatin-induced apoptosis was determined.

**Results:**

These prostate and colon cancer cell lines were initially TC-NER proficient and RNAi against CSB significantly reduced their DNA repair capacity. Decreased TC-NER capacity was associated with an increase in the sensitivity of tumour cells to cisplatin-induced apoptosis, even in p53 null and DNA mismatch repair-deficient cell lines.

**Conclusion:**

The present work indicates that CSB and TC-NER play a prominent role in determining the sensitivity of tumour cells to cisplatin even in the absence of p53 and DNA mismatch repair. These results further suggest that CSB represents a potential target for cancer therapy that may be important to overcome resistance to cisplatin in the clinic.

## Background

Cisplatin [*cis*-diammine-dichloroplatinum (II)] has been used in the treatment of neoplastic diseases for over 30 years [1]. The effectiveness of cisplatin is dependent on its interaction with DNA. This drug forms a variety of DNA adducts but more than 99% of these are intrastrand DNA adducts, most often between adjacent purines, with only a very small number of interstrand-crosslinks and monoadducts [2]. These intrastrand lesions are repaired by the nucleotide excision repair (NER) pathway so the response of tumours to cisplatin and other platinum-based drugs may be affected by nucleotide excision repair capacity of the tumour cells [1].

The vast majority of what is known about NER stems from studies using the model DNA damaging agent UV light but NER of cisplatin-induced DNA adducts is thought to occur through an identical mechanism [3-8]. The rate-limiting step in NER is lesion recognition and this occurs through two distinct mechanisms yielding two interrelated yet genetically separable subpathways of NER [9]. Global-genomic nucleotide excision repair (GG-NER) is responsible for the removal of the vast majority of UV and cisplatin-lesions throughout the genome whereas transcription-coupled nucleotide excision repair (TC-NER) is responsible for the selective removal of only those lesions that are present in the template strand of expressed genes [3,5,10-12].

Cockayne syndrome (CS) and xeroderma pigmentosum (XP) are heterogeneous disorders characterized by clinical photosensitivity [13]. Based on cell fusion and complementation studies, patients with CS were classified into two groups (CS groups A and B) whereas XP patients were grouped into 8 groups (XP groups A through G and V). CS and XP cells (with the exception of the variant form, XP-V) have defects in NER. These defects can be specific to TC-NER, GG-NER or both sub-pathways of NER [14,15]. Therefore, fibroblasts derived from patients with these UV sensitive syndromes have been instrumental in identifying proteins involved specifically in TC-NER and GG-NER and provided a model system to study the relative contribution of GG-NER and TC-NER to cisplatin response [6,16-18].

All TC-NER-deficient (CS-B for example) and completely NER-deficient (XP-A for example) fibroblasts were found to be exquisitely sensitive to apoptosis induced by UV light and cisplatin [6,16-18]. Many genetic alterations in cancer affect DNA damage responses. Notably, loss of the p53 tumour suppressor protein and DNA mismatch repair (MMR) proteins are among the most common genetic alterations in cancer and these alterations have been associated with resistance to cisplatin treatment [19-27]. Therefore, the effect of targeting TC-NER on the acute response of tumour cells to cisplatin could not be accurately predicted.

Here we report that silencing CSB by RNA interference (RNAi) reduced the TC-NER capacity of several prostate and colon cancer cell lines. This repair defect was associated with increased sensitivity of CSB-targeted cells to cisplatin-induced apoptosis. Importantly, the sensitivity of p53- and/or MMR-deficient tumour cells to cisplatin-induced apoptosis could be significantly increased by silencing CSB. These results suggest that TC-NER plays a major role in determining the sensitivity of these tumour cells to cisplatin and further suggests that CSB represents a potential therapeutic target for cancer therapy.

## Methods

### Cell Culture and UV-irradiation

HCT116, DU145 and PC-3 cells were obtained from the American Tissue Type Collection (Camden, NJ). The MLH1-corrected (HCT116 + chr3) and p53 nullizygous (HCT116p53-/-) cell lines were described previously [28,29]. HCT116 derived cells were cultured in McCoy's media (Wisent, St. Bruno, QC) while DU145 and PC-3 cells were grown in DMEM (Hyclone, Logan, UT). McCoy's and DMEM were supplemented with 10% fetal bovine serum (Wisent, St. Bruno, QC). Where indicated, cisplatin (Mayne Pharma Canada Inc., Montreal, QC) was added to fresh, pre-warmed media at the indicated final concentration.

To UV-irradiate cells, medium was removed and cells were irradiated with the indicated dose using a germicidal bulb emitting predominantly at 254 nm at 1 J/m^2^/s as measured with a hand-held UV dosimeter (UVX Radiometer, UVP Inc., Uplands, CA). Fresh, pre-warmed media was replaced and dishes were returned to an incubator for the indicated period of time.

### RNA interference

Sub-confluent cells were transfected with the indicated siRNA (Dharmacon, Lafayette, CO) using OptiMEM II and Oligofectamine (Invitrogen, Burlington, ON). The target sequences for CSB and XPA were GTGTGCATGTGTCTTACGA and AGAATTGCGGCGAGCAGTA, respectively. These RNA duplexes were used at a final concentration of 50 nM. A non-targeting control siRNA (TAGCGACTAAACACATCAA) was used as a negative control.

### Preparation of nuclear lysates

Cells were rinsed with phosphate buffered saline (PBS) then trypsinized and collected by centrifugation. Cell pellets were resuspended in nuclear extraction buffer (320 mM sucrose, 10 mM HEPES, 5 mM MgCl_2_, 1% triton-X-100, pH 7.4), incubated on ice and then collected by centrifugation at 2500 × g. The resulting pellets were rinsed twice with nuclear wash buffer (320 mM sucrose, 10 mM HEPES, 5 mM MgCl_2_, pH 7.4) and collected by centrifugation at 2500 × g then resuspended in RIPA buffer (50 mM Tris-HCl pH6.8, 150 mM NaCl, 1 mM EDTA, 1% Triton-X-100, 1% sodium deoxycholate, 0.1% SDS). Pellets were disrupted using a sonicator equipped with a chilled microtip (Thermo Fisher Scientific, Ottawa, ON) and protein quantified using the Bradford assay (Bio-Rad, Mississauga, ON).

### Immunoblotting

Two hundred micrograms of nuclear protein per well was subjected to gel electrophoresis using NuPAGE 3-8% gradient polyacrlyamide gels (Invitrogen), to visualize CSB protein whereas MLH1 and Ku86 were resolved using NuPAGE 4-12% gradient polyacrylamide gels (Invitrogen). Proteins were transferred to Hybond-C nitrocellulose (GE Healthcare, Baie d'Urfé, QC) and blots were stained with Ponceau S Red (5 mg/ml Ponceau S Red, 2% glacial acetic acid) to visualize total transferred proteins. Blots were then blocked in PBSMT-A (PBS, 5% nonfat milk powder, 0.05% Tween 20) proteins were detected using antibodies against XPA (FL-273), CSB (E-18) and Ku86 (M-20) (Santa Cruz Biotech, Santa Cruz, CA) and against MLH-1 (clone G168-15, BD Biosciences, Mississauga, ON) diluted in PBSMT-B (PBS, 0.5% nonfat milk powder) and were visualized using SuperSignal West Pico Chemiluminescent Substrate (Thermo Fisher Scientific) in combination with X-ray film (Kodak, Rochester, NY). Multiple proteins were detected using the same blots using Restore Western Blot Stripping Buffer (Thermo Fisher Scientific).

### The recovery of RNA synthesis

Sub-confluent cells were transfected with siRNA and subsequently grown in media supplemented with 5 μCi/mL of [^14^C] thymidine (GE Healthcare) to uniformly label DNA. Seventy-two hours later, cells were treated with 10 J/m^2 ^UV. One hour before indicated collection time, the media was replaced with fresh media containing 50 μCi/mL [^3^H] uridine (GE Healthcare) to label nascent RNA. Samples were rinsed in PBS containing 0.2% sodium azide (PBS-Z), collected by trypsin in PBS-Z, rinsed with PBS-Z and cell pellets were stored at -80°C. Samples were lysed in 1% SDS and nucleic acids were precipitated in 10% trichloroacetic acid (TCA)/0.1 M sodium pyrophosphate (NaPPi) and precipitated nucleic acids were collected on glass fiber filters (Thermo Fisher Scientific). Incorporation of [^3^H] and [^14^C] was determined using a scintillation counter and [^3^H] counts were normalized to [^14^C] counts to control for cell number. RNA synthesis is expressed as the proportion of [^3^H] uridine incorporated in UV-treated samples compared to unirradiated controls.

### Host cell reactivation

Recombinant adenovirus expressing the *lacZ *gene under control of the murine cytomegalovirus promoter [30], was suspended in a minimal volume of PBS and was subsequently irradiated with the indicated dose of UV light on ice, as previously described [31]. Cells were infected with UV- or mock-treated AdlacZ at a multiplicity of infection of 5 plaque forming units per cell. Forty-eight hours following infection, media was removed and monolayers were incubated with 1 mM chlorophenolred-β-D-galactopyranoside (Fluka Biochemika, Buchs, Switzerland) in 0.01% Triton X-100, 1 mM MgCl_2_, and 100 mM phosphate buffer (pH 8.3) [32]. Absorbance at 570 nm was determined using a Thermo Multiskan Ascent microplate photometer (Thermo Fischer Scientific). β-galatosidase activity from the indicated dose is expressed relative to the activity obtained by infection with un-irradiated virus.

### Flow cytometry

Cells were treated with the indicated dose of UV light or cisplatin, 72 hours following transfection of the indicated siRNA. Detached and adherent cells were collected 48 hours following treatment, fixed in 70% ethanol for a minimum of 2 hours at -20°C and stained in 30 μM propidium iodide (Sigma-Aldrich, Oakville, ON) in PBS with 40 μg/mL of RNAse A (Sigma-Aldrich) [33]. Samples were analyzed by fluorescence activated cell sorting using a Becton Dickenson LSR II Facstation and CellQuest software (Becton Dickinson, Franklin Lakes, NJ) and data files were analyzed using FCS Express (De Novo Software, Los Angeles, CA). Apoptosis was quantified as the proportion of cells with subdiploid DNA content.

### Caspase activity assays

Cells were transfected with the indicated siRNA and treated with either UV light or cisplatin. Twenty four hours following treatment, cells were collected with trypsin, rinsed thoroughly with PBS and cell number was determined using an automated cell counter (Vi-Cell XR, Beckman Coulter). Caspase 3, 8 and 9 activities were determined from 1 × 10^6 ^cells using ApoAlert caspase-3, caspase-8 or caspase-9/6 Fluorescent Assay Kits as specified by the manufacturer (Clontech, Mountain View, CA). Fluorescence was measured using a Thermo Fluoroskan Ascent microplate fluorometer (Thermo Fisher Scientific).

## Results

### RNA interference against CSB increased the sensitivity of prostate cancer cells to cisplatin-induced apoptosis

**Figure 1:**
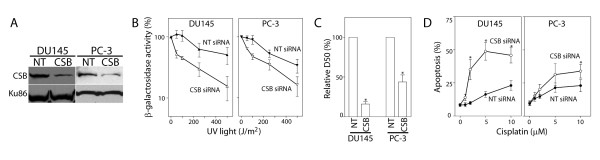
**RNA interference in prostate cancer cell lines**. (A) DU145 and PC-3 cells were transfected with non-targeting control (NT) or anti-CSB (CSB) siRNA and CSB levels were assessed by immunoblot analysis of nuclear lysates. CSB and Ku86 migrated with apparent molecular weights of approximately 175 and 85 kDa, respectively. (B) Host cell reactivation of a UV-damaged reporter gene was assessed in DU145 and PC-3 cells transfected with non-targeting control (NT) or anti-CSB (CSB) siRNA. (C) The data in (B) is expressed as the dose required to reduce β-galactosidase activity by 50%. (D) The indicated cell lines were transfected with control (NT- closed symbols) or CSB targeting (open symbols) siRNA. Apoptosis was assessed as the proportion of cells with sub-diploid DNA content 48 hours following exposure to the indicated dose of cisplatin. Each value in B-D represents the mean (± SEM) from at least 4 independent experiments. An * in C indicates that the value is significantly less than 100% (P ≤ 0.05, single sample t-test) while an * in D indicates that the value is significantly different than its respective NT control (P ≤ 0.05, t-test). Similar results were obtained following exposure to UV light (data not shown).

Prostate cancer cells (DU145 and PC-3 cells) were transfected with control or anti-CSB siRNAs and CSB protein levels were assessed by immunoblot analysis (Figure 1A). A single UV-induced dimer in the template strand of an active gene is sufficient to block its expression [34-36]. This principal forms the fundamental basis of host cell reactivation (HCR) assay that is commonly used to measure the repair of transcription-blocking DNA lesions [31,36-38]. The DU145 and PC-3 cells exhibited a similar dose-dependent decrease in β-galactosidase activity, suggesting that these cell lines have a similar capacity to repair transcription-blocking UV lesions (Figure 1B). Transfection of siRNAs directed against CSB led to a significant decrease in HCR of the UV-damaged reporter gene in both prostate cancer cell lines (Figure 1B). The dose of UV light required to reduce β-galactosidase activity to 50% (D50) was significantly lower in CSB targeted cells compared to control siRNA transfected cells (Figure 1C). Therefore, silencing CSB by RNAi resulted in impaired TC-NER, allowing us to assess the contribution of TC-NER to cisplatin response in these tumour cells.

The expression of CSB was similarly inhibited by RNAi and the sensitivity of PC-3 and DU145 cells to cisplatin-induced apoptosis was assessed. Silencing CSB led to a large increase in the sensitivity of DU145 cells to cisplatin-induced apoptosis (Figure 1D). The increased sensitivity of PC-3 cells was evident albeit less pronounced (Figure 1D). These results indicate that the ability of these prostate cancer cell lines to repair transcription-blocking DNA lesions by TC-NER was significantly reduced when CSB levels were decreased by RNAi. Importantly, these results suggest that disruption of CSB may be beneficial in combination with platinum-based drugs in the management of prostate cancer.

### Role of p53 and MLH1 in determining cisplatin response of CSB-targeted colon cancer cells

Tumour cells frequently express mutant forms of DNA damage response proteins that may influence their sensitivity to therapeutic agents like cisplatin. For example, loss of p53 and MMR have been associated with resistance to cisplatin [19-23,25]. Notably, DU145 cells carry point mutations in both alleles of p53 and do not express MLH1 so they are MMR- and p53-deficient [39,40]. Similarly, PC-3 cells do not express detectable p53 and they exhibit microsatellite instability indicative of a defect in MMR [39,40]. Given the effect of targeting CSB in these p53 and MMR-deficient prostate cancer cell lines, an isogenic series of cell lines derived from HCT116 colorectal cancer cells were used to better assess the impact of p53- and MMR-deficiency under conditions in which CSB is silenced by RNAi.

**Figure 2:**
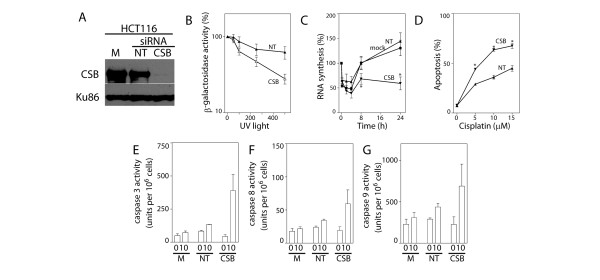
**Silencing CSB in HCT116 cells**. (A) The effectiveness of siRNAs against CSB in HCT116 cells was assessed by immunoblot analysis of nuclear lysates from cells that were either mock-transfected (M) or transfected with the indicated siRNA (non-targeting control (NT) or CSB). (B) Host cell reactivation of a UV-damaged reporter gene was assessed in non-targeting siRNA (NT) and CSB siRNA (CSB) transfected cells. Reduced CSB levels were associated with a decrease in the dose required to reduce β-galactosidase activity to 50% (P < 0.05, t-test). (C) The ability of mock-, NT- and CSB-siRNA transfected cells to recover nascent RNA synthesis following UV exposure was assessed in HCT116 cells. (D) Similarly transfected HCT116 cells were exposed to the indicated dose of cisplatin and apoptosis was assessed as the proportion of cells with subdiploid DNA content 48 hours later. (E-G) The activity of caspases 3, 8 and 9, respectively, was determined 24 hours following exposure to 10 μM cisplatin. Each value in B-G represents the mean (± SEM) determined from a minimum of 3 independent experiments. An * in C indicates that the value is significantly less than 100% (P ≤ 0.05, single sample t-test) while an * in D indicates that the value is significantly different from its respective NT control (P ≤ 0.05, t-test). There was a significant difference in caspase 3 but not caspases 8 or 9 activity among transfectants following cisplatin treatment (P = 0.04, 0.14 and 0.30, respectively, ANOVA).

HCT116 cells express wildtype p53 but do not express MLH1 and are considered MMR-defective [28]. The anti-CSB siRNA reduced CSB protein levels by more than 90% in these cells (Figure 2A) and this led to a decrease in their ability to repair an UV-damaged reporter gene (Figure 2B). Specifically, the dose of UV light required to reduce β-galactosidase activity to 50% (D50) was significantly lower in CSB-targeted HCT116 cells compared to control siRNA transfected cells. As a second measure of TC-NER, we assessed the ability of cells to recover nascent RNA synthesis following UV exposure. Whereas mock and control siRNA transfected cells recovered nascent RNA synthesis within 8 hours following exposure to 10 J/m^2 ^of UV light, the incorporation of [^3^H] uridine was still significantly inhibited 24 hours following UV exposure of CSB siRNA transfected HCT116 cells (Figure 2C). Taken together, silencing CSB led to a reduction in the capacity of HCT116 cells to perform TC-NER.

HCT116 were transfected with control and anti-CSB siRNAs and their sensitivity to cisplatin-induced apoptosis was assessed. Consistent with the results in prostate cancer cell lines, targeting CSB in HCT116 cells increased their sensitivity to cisplatin-induced apoptosis (Figure 2D) and this was associated with increased caspase activity (Figures 2E-G). Taken together, silencing CSB by RNAi reduced the capacity of these MMR-deficient cells to repair transcription-blocking DNA lesions and greatly increased their sensitivity to cisplatin-induced apoptosis.

**Figure 3:**
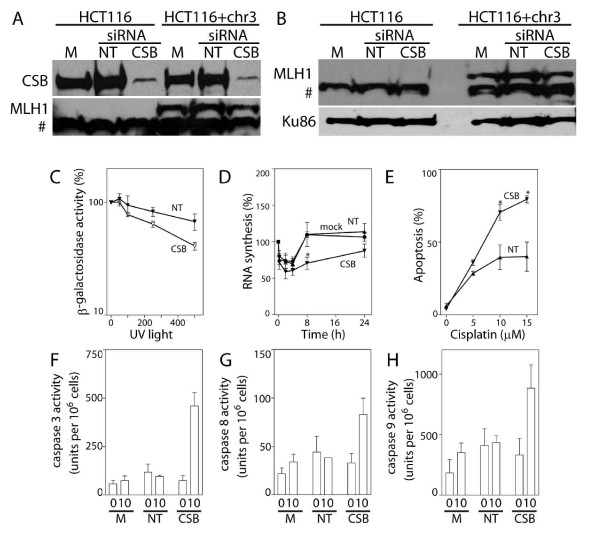
**RNAi against CSB in DNA mismatch repair corrected HCT116 cells**. (A) The effectiveness of siRNAs against CSB in HCT116 and HCT116 + chr3 cells was assessed by immunoblot analysis of nuclear lysates from cells that were either mock-transfected (M) or transfected with the indicated siRNA (non-targeting control (NT) or CSB). HCT116 + chr3 cells express readily detectable MLH1 protein (A and B). The # denotes a non-specific band recognized by this antibody. (C) Host cell reactivation of a UV-damaged reporter gene was determined in non-targeting siRNA (NT) and CSB siRNA (CSB) transfected HCT116 + chr3 cells. Reduced CSB levels were associated with a decrease in the dose required to reduce β-galactosidase activity to 50% (P < 0.05, t-test). (D) The ability of mock-, NT- and CSB-siRNA transfected HCT116 + chr3 cells to recover nascent RNA synthesis following UV exposure was assessed in HCT116 + chr3 cells. (E) Similarly transfected HCT116 + chr3 cells were exposed to the indicated dose of cisplatin and apoptosis was assessed as the proportion of cells with subdiploid DNA content 48 hours later. (F-H) The activity of caspases 3, 8 and 9 was determined 24 hours following exposure to 10 μM cisplatin. Each value in C-H represents the mean (± SEM) determined from a minimum of 3 independent experiments. An * in D indicates that the value is significantly less than 100% (P ≤ 0.05, single sample t-test) while an * in E indicates that the value is significantly different from its respective NT control (P ≤ 0.05, t-test). There was a significant difference in caspase 3, 8 and 9 activity among transfectants following cisplatin treatment (P = 0.001, 0.03 and 0.05, respectively, ANOVA). Similar results were obtained following exposure to UV light (data not shown).

Similar experiments were performed in an MLH1-corrected MMR-proficient subline of HCT116 cells (HCT116 + chr3) (Figure 3A and 3B) [28]. Again, targeting CSB by RNAi in these cells inhibited HCR of the UV-damaged reporter gene and prevented the efficient recovery of nascent RNA synthesis (Figures 3C and 3D). Furthermore, RNAi against CSB increased the sensitivity of CSB-targeted cells to cisplatin-induced apoptosis and this was associated with significant increases in the activity of caspases 3, 8 and 9 (Figures 3E-H). Taken together, MLH1 had no apparent effect on TC-NER of UV-induced DNA lesions or the sensitivity of these cells to cisplatin-induced apoptosis. Targeting CSB by RNAi was equally effective at increasing the sensitivity of these tumour cells to cisplatin-induced apoptosis, regardless of MLH1 expression.

**Figure 4:**
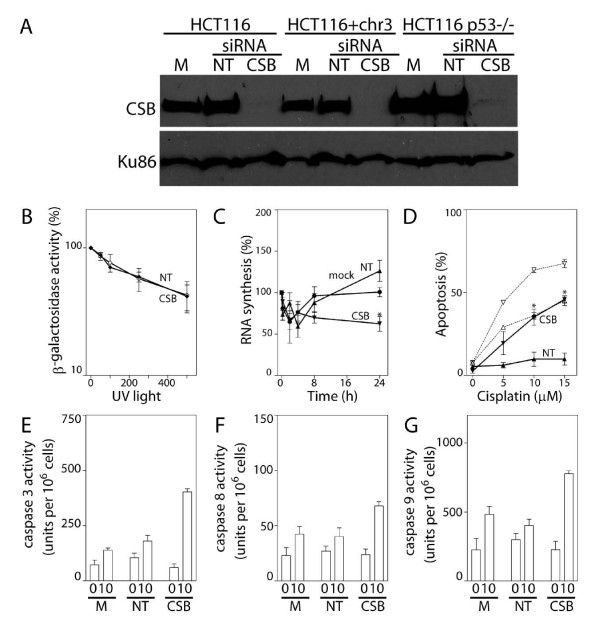
**RNAi against CSB in HCT116p53-/- cells**. (A) The effectiveness of siRNAs against CSB in HCT116, HCT116 + chr3 and HCT116p53-/- cells was assessed by immunoblot analysis of nuclear lysates from cells that were either mock-transfected (M) or transfected with the indicated siRNA (non-targeting control (NT) or CSB). (B) HCR of a UV-damaged reporter gene was determined in non-targeting siRNA (NT) and CSB siRNA (CSB) transfected HCT116p53-/- cells. (C) The ability of mock-, NT- and CSB-siRNA transfected cells to recover nascent RNA synthesis following UV exposure was assessed in HCT116p53-/- cells. (D) The sensitivity of HCT116p53-/- cells (closed symbols, solid lines) transfected with NT (triangles) or CSB (inverted triangles) siRNAs to cisplatin-induced apoptosis was assessed, as described in figure 2D. The results of similar experiments presented in figure 2D for HCT116 cells (open symbols, dashed lines) are provided for direct comparison. (E-G) The activity of caspases 3, 8 and 9, was determined following exposure to 10 μM cisplatin. Each value in B-G represents the mean (± SEM) determined from a minimum of 3 independent experiments. An * in C indicates that the value is significantly less than 100% (P ≤ 0.05, single sample t-test) while an * in D indicates that the value is significantly different from its respective NT control (P ≤ 0.05, t-test). A significant difference in caspase 3, 8 and 9 activity was detected among transfectants following cisplatin treatment (P = 0.0001, 0.04 and 0.002, respectively, ANOVA). Similar results were obtained following exposure to UV light (data not shown).

CSB was also silenced by RNAi in p53 nullizygous derivatives of HCT116 cells (HCT116p53-/- cells) (Figure 4A). These p53 null cells appeared to be reduced in their capacity to repair the UV damaged reporter gene compared to parental cells already and HCR of the UV-damaged reporter gene was not further reduced by siRNA against CSB (Figure 4B). Subtle defects in TC-NER have been reported using similar HCR assays in other p53-deficient cells [41]. Nonetheless, these cells had almost fully recovered nascent RNA synthesis within 8 hours following UV exposure and this was again delayed in CSB siRNA-transfected HCT116p53-/- cells (Figure 4C). Despite the apparent repair defect detected in the HCR experiments, the present results indicate that HCT116 p53-/- cells are not fully TC-NER deficient because RNAi against CSB again abrogated the recovery of nascent RNA synthesis in these cells.

There was no significant increase in the proportion of control siRNA transfected HCT116p53-/- cells undergoing apoptosis following exposure to up to 15 μM of cisplatin indicating that these cells were relatively resistant to cisplatin-induced apoptosis compared to isogenic controls (Figure 4D). This is likely due to the disruption of the well-described pro-apoptotic activity of p53 in these nullizygous cells [42]. However, decreased expression of CSB was again associated with a significant increase in the sensitivity of tumour cells to cisplatin-induced apoptosis (Figure 4D). Apoptosis was associated with a significant increase in the activity of caspases 3, 8 and 9 (Figure 4E-G). So, while p53 nullizygous cells were less sensitive to cisplatin-induced apoptosis, targeting TC-NER was similarly effective at sensitizing HCT116p53-/- cells to cisplatin-induced cell death. Clearly, p53 was not absolutely required to sensitize tumour cells to cisplatin when CSB expression was decreased by RNAi.

### RNA interference against XPA sensitizes cancer cells to cisplatin

**Figure 5:**
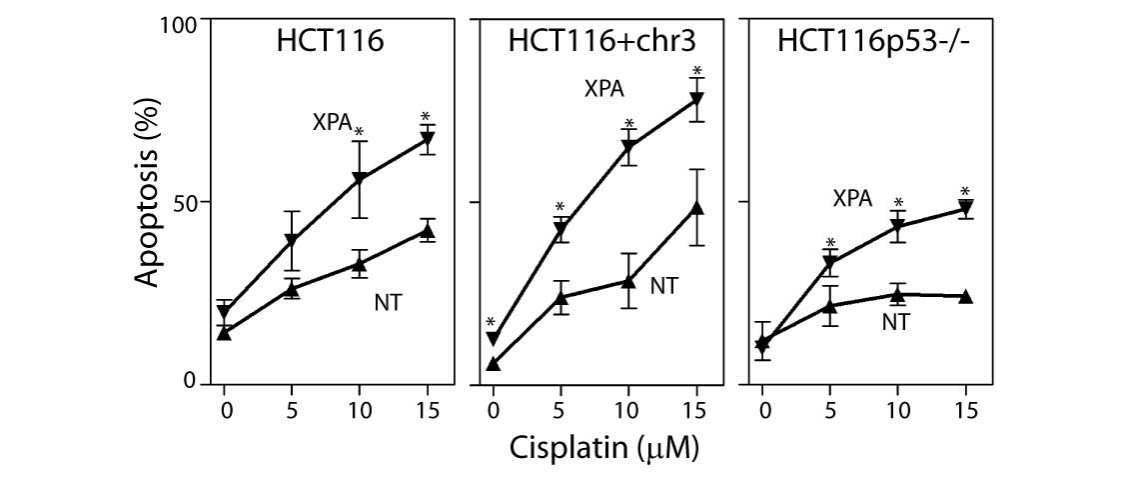
**RNAi against XPA sensitizes colon cancer cells to cisplatin**. HCT116, HCT116 + chr3 and HCT116p53-/- cells were transfected with non-targeting control (NT) or XPA siRNA. These transfected cells were exposed to the indicated concentration of cisplatin and apoptosis was assessed 48 hours later by the proportion of cells with subdiploid DNA content. Each value represents the mean (± SEM) determined from a minimum of 3 independent experiments. An * indicates that the value is significantly different its respective NT control (P ≤ 0.05, t-test). Similar results were obtained following exposure to UV light (data not shown).

The CS proteins have been known to participate in TC-NER for many years [14], however, the CS proteins may play an additional role in regulating transcription [43-45]. Therefore, the expression of another protein required for TC-NER was silenced by RNAi. XPA is a DNA damage binding protein that is required for both TC-NER and GG-NER to which no additional functions have been ascribed [46]. XPA protein levels were reduced by RNAi in HCT116, HCT116 + chr3 and HCT115p53-/- cells and the sensitivity of the targeted cells to cisplatin-induced apoptosis was assessed. Decreased expression of XPA was associated with a statistically significant increase in the sensitivity of all 3 cell lines to cisplatin-induced apoptosis (Figure 5). Taken together, decreased expression of CSB or XPA was associated with increased sensitivity of tumour cells to cisplatin-induced apoptosis and this was largely independent of MMR and p53.

## Discussion

### Link between nucleotide excision repair and sensitivity to cisplatin

The contribution of TC-NER to cell survival following UV-irradiation or cisplatin treatment is thought to be cell type specific. For example, CS-B fibroblasts have a specific defect in TC-NER and are more sensitive to UV- and cisplatin-induced apoptosis than GG-NER deficient XP-C fibroblasts [6,16-18]. A similar relationship was reported in CSB nullizygous mouse embryonic fibroblasts and murine keratinocytes exposed to UV light [7,16,17,47-53]. By contrast, CSB null murine embryonic stem cells were not hypersensitive to this DNA damaging agent [52]. The cell type-specific roles for TC-NER in determining the fate of UV-irradiated cells raised the question as to whether the response of tumour cells to UV light and cisplatin would more closely resemble that of primary cells [7,16-18,33,47-49,54,55] or embryonic stem cells [52].

The CSB protein is required for a rate limiting step of TC-NER [56-58]. Here we show that a series of prostate and colon cancer cell lines retained the ability to repair transcription-blocking DNA lesions and that silencing CSB functionally impaired this repair process. Therefore, we were able to test the role of TC-NER in cisplatin response using this *in vitro *system. Decreased expression of CSB was associated with increased sensitivity of cells to cisplatin-induced apoptosis. Similarly, silencing CSB led to increased apoptosis following exposure to UV light (data not shown), consistent with a role for CSB in determining the sensitivity of cells to apoptosis induced by both of these agents [6,16-18,50,59,60]. Lu and coworkers reported that antisense oligonucleotides designed to inhibit the expression of CSB increased the responsiveness of wildtype p53 and DNA mismatch repair proficient ovarian tumour xenografts to cisplatin [61]. Targeting TC-NER may represent a means of increasing the responsiveness of a variety of tumours to cisplatin.

### MMR proteins and p53 in cisplatin resistance

Cisplatin is among the most widely used anti-neoplastic agents. Although tumours often respond favorably to cisplatin treatment, there is eventual disease progression in many cases. This recurrent disease can be refractory to subsequent treatment with platinum-based drugs [1,62-69]. Drug resistance can in some cases be attributed to increased DNA repair response but may also result from a variety of other alterations including decreased apoptotic signaling in response to this form of DNA damage [64,67,69-74]. Two key genetic changes that have been associated with resistance to cisplatin are p53- and MMR-deficiency [19,21-23,25,64,75-77]. Despite the association of p53 and MMR defects with cisplatin resistance, RNAi against CSB and XPA in this panel of tumour cell lines deficient in p53 (PC-3, DU145 and HCT116p53-/-) and MMR (PC-3, DU145, HCT116 and HCT116p53-/-) resulted in a significant increase in the sensitivity of cells to cisplatin-induced cell death. The present results indicate that targeting TC-NER sensitizes tumour cells to cisplatin-induced apoptosis and that this was largely independent of p53 and MMR. This makes TC-NER an attractive target for combined cancer therapy.

## Conclusion

We found that both p53 and MMR-deficient colorectal and prostate cancer cells retain the ability to perform TC-NER and targeting TC-NER in these cells increased their sensitivity to UV light and cisplatin. CSB and XPA may represent rational targets to augment cisplatin responsiveness of tumours, independent of MMR capacity and p53. This work further suggests that targeting other proteins involved in TC-NER may similarly represent a promising approach for cancer therapy. Notably, recent clinical evidence suggests that ERCC1 levels predict response to platinum-based therapies in non-small cells lung cancer [1,78-80], small cell lung cancer [81], esophageal cancer [82], head and neck cancer [83], bladder cancer [84] and testicular cancer[85]. ERCC1, like XPA, is required for both TC-NER and GG-NER. It is possible that the predictive value of ERCC1 is related to its role in TC-NER and that the expression of other proteins involved in TC-NER, like CSB, may also be predictors of therapeutic response in diverse tumours.

## Competing interests

The authors declare that they have no competing interests.

## Authors' contributions

LJS and BCM conceived of the study. LJS and JMS collected the data. LJS and BCM contributed to data analysis and wrote the manuscript with input from JMS. All authors read and approved the manuscript.

## Pre-publication history

The pre-publication history for this paper can be accessed here:

http://www.biomedcentral.com/1471-2407/10/207/prepub
